# Differential immune responses to SFTSV Gn and Gc mRNA vaccines in mice and dogs

**DOI:** 10.1128/spectrum.03603-25

**Published:** 2026-07-13

**Authors:** Zezheng Jiang, Li Tian, Zhongxin Zhao, Xinyu Wu, Lele Liu, Menghua Li, Hua Qian, Chunhai Gao, Xiaoying Lei, Wenwen Zheng, Xuejie Yu, Xuexing Zheng

**Affiliations:** 1 Department of Virology, School of Public Health, Cheeloo College of Medicine, Shandong University 12589, Jinan, Shandong, China; 2 Department of Laboratory Medicine, Linyi People’s Hospital, Linyi, Shandong, China; 3 Cheeloo College of Medicine, Shandong University, The Second Hospital, Jinan, Shandong, China; 4 State Key Laboratory of Virology, School of Public Health, Wuhan University, Wuhan, Hubei, China; Konkuk University, Seoul, Republic of Korea

**Keywords:** severe fever with thrombocytopenia syndrome virus, Gn/Gc protein, neutralizing antibody, mRNA vaccine, immune response

## Abstract

Severe fever with thrombocytopenia syndrome virus (SFTSV) is a tick-borne zoonotic pathogen with high mortality and no licensed vaccines. We developed two lipid nanoparticle-encapsulated mRNA vaccines encoding the Gn and Gc glycoproteins of SFTSV and evaluated their immunogenicity and protective efficacy in mice, as well as their immunogenicity in dogs. In mice, the Gn mRNA vaccine induced higher neutralizing antibody titers and stronger cellular immune responses than the Gc vaccine, and both conferred complete protection against lethal SFTSV challenge in IFNAR-blocked mice. In contrast, in dogs, the Gc mRNA vaccine elicited significantly higher neutralizing antibody titers than Gn, despite comparable safety profiles. This species-dependent divergence in immunogenicity underscores the importance of host-specific considerations in antigen selection for zoonotic vaccine development. These findings support the immunogenic potential of both constructs as candidate vaccines against SFTSV.

**IMPORTANCE:**

In this study, we developed mRNA vaccines targeting the severe fever with thrombocytopenia syndrome virus Gn and Gc glycoproteins separately and systematically evaluated their immunogenicity in murine and canine models. Our findings demonstrate that both vaccine formulations effectively elicited potent neutralizing antibody responses in immunized animals. Notably, we observed distinct species-specific immunogenicity patterns: the Gn-targeted vaccine showed superior performance in a murine model, whereas the Gc-targeted variant induced more robust immune activation in beagles. These results emphasize that vaccine selection must account for species-specific efficacy.

## INTRODUCTION

Severe fever with thrombocytopenia syndrome (SFTS) is a rapidly emerging zoonosis caused by severe fever with thrombocytopenia syndrome virus (SFTSV). In 2024, the World Health Organization (WHO) listed SFTSV among the priority pathogens with pandemic potential, underscoring its capacity to breach species barriers and circulate in both humans and animals. SFTSV was first reported in China in 2011, with cases primarily distributed in rural areas of central and eastern China (1). SFTS cases have subsequently been reported in Japan, Myanmar, South Korea, Thailand, and Vietnam (2–6). The clinical manifestations of SFTS primarily include fever, fatigue, leukopenia, thrombocytopenia, hemorrhagic symptoms, gastrointestinal disturbances, and multiorgan failure, with fatality rates ranging from 12% to 30% (7), and the incidence has shown an increasing trend in recent years (8). The incidence of SFTS is predominantly observed between April and October each year, especially the highest incidence from May to July, with obvious seasonal characteristics. Epidemiological investigations revealed that 90% of the patients were over 40 years old, and the case fatality rates tended to increase with age (9).

SFTSV, which was named *Bandavirus dabieense* by the International Committee on Taxonomy of Viruses in 2024, belongs to the genus *Bandavirus*, family Phenuiviridae, order Hareavirales, and class Bunyaviricetes. SFTSV is an enveloped RNA virus whose genome consists of three single-stranded negative-sense RNA fragments (*L*, *M*, and *S*), encoding RNA-dependent RNA polymerase, glycoproteins (GPs) (Gn/Gc), nucleoprotein, and nonstructural protein, respectively. The Gn protein is a type I transmembrane protein that encodes neutralizing antibody epitopes that are capable of binding neutralizing antibodies in the host. The Gc protein is a type II transmembrane protein that plays a crucial role in receptor binding and membrane fusion, although its protective role has been less extensively studied (10–12). SFTSV is transmitted primarily by ticks, and *Haemaphysalis longicornis* is considered the main vector tick. With the changing global climate, the number of regions suitable for the survival of *Haemaphysalis longicornis* is gradually increasing, which also suggests that the potential range of the impact of SFTSV may be expanding (13). However, although *Haemaphysalis longicornis* is the principal vector, SFTSV infection is not confined to arthropod-human cycles (14–16). Viral RNA or serological evidence has been detected in a wide range of animals, including livestock (e.g., dogs, cats, goats, pigs, cattle, deer) and wildlife (rodents and avian species) (17–19). Of particular concern is the infection in companion animals, notably dogs and cats, which exhibit higher mortality rates than humans do (20, 21). Several investigations have documented probable dog-to-human and cat-to-human transmission after close contact with viremic pets, including veterinary clinical outbreaks and household clusters without tick exposure (22–24). These observations confirm that the SFTSV can move bidirectionally across the animal-human interface, greatly complicating control efforts.

Currently, 92% of SFTS patients are farmers engaged in long-term agricultural work who reside in wooded/hilly areas and have close contact with natural environments (25–27). As a high-risk population for SFTSV infection (27), they often lack access to appropriate medical support. Given that SFTS is a viral infectious disease with a relatively high fatality rate and that no specific drugs are currently available, preventive measures against this disease are essential. To align with the WHO’s Immunization Agenda 2030 (28), it is necessary to provide immunization protection for such high-risk populations. Furthermore, studies have identified SFTSV-carrying ticks in urban parks, which may transmit the virus to humans through direct bites or pet-mediated transmission (29–31). Nevertheless, there is currently no approved vaccine to prevent SFTSV infection effectively.

mRNA vaccines combine rapid, cell-free manufacturing with potent humoral and cellular immunity (32, 33). Given the recognized role of dogs as both sentinel and potential amplifier hosts for SFTSV, we hypothesized that a vaccine capable of eliciting robust immune responses in dogs would both (i) mitigate the risk of pet-to-human spillover and (ii) provide translational insight for human vaccination. In this study, we designed two mRNA vaccines on the basis of the coding sequences of SFTSV Gn and Gc. The designed signal peptide ensures the correct expression of the downstream target protein. The immunogenicity of the two vaccines was evaluated at both the cellular and animal levels, and the ability of Gn and Gc to induce specific neutralizing antibodies and protection rates was compared.

## MATERIALS AND METHODS

### Viruses and cells

The SFTSV JS2011-013-1 strain was used in this study. Vero and HEK293T cells, both of which were cultured in Dulbecco’s modified Eagle’s medium (DMEM; Gibco) supplemented with 10% fetal bovine serum (FBS; Gibco) and 1% penicillin/streptomycin at 37°C with 5% CO_2_, were obtained from the Microbiology Laboratory at Shandong University.

### Animals

Wild-type BALB/c mice (6–8 weeks) were purchased from Jinan Pengyue Experimental Animal Co., Ltd., and beagles were purchased from Jinan Xilingjiao Breeding and Cultivation Center. Animals were placed in controlled environments (12 h light/dark cycle; 20°C–26°C; 40%–60% humidity) and had free access to bacteria-free water and food. For animal research, all mice/dogs were randomly divided into three groups using a random number table: control, lipid nanoparticle (LNP)-mRNA-Gn, and LNP-mRNA-Gc. The mice in each group were sacrificed by cervical dislocation.

### Reagents

Lipofectamine 3000 Reagent was purchased from Thermo Fisher Scientific. Phosphate-buffered saline (PBS) was purchased from Macgene (CC008). The mouse anti-SFTSV Gn monoclonal antibody and human anti-SFTSV N monoclonal antibody (34) were generously provided by Professor Xuejie Yu from Wuhan University. The rabbit anti-Gc polyclonal antibody and mouse anti-SFTSV Gc polyclonal antibody were retained in our laboratory. The mouse anti-GAPDH monoclonal antibody was purchased from Proteintech (60004-1-Ig). The HIScribe T7 RNA synthesis kit, vaccinia virus capping enzyme, mRNA cap 2′-O-methyltransferase, *Escherichia coli* poly(A) polymerase, and Monarch RNA purification kit were all purchased from New England Biolabs (Shanghai) Co., Ltd. The tribromoethanol (30 μL/g) used for anesthesia was purchased from MeilunBio (75-80-9). ColorMixed Protein Marker 250 was purchased from ABclonal (RM02949). HRP-conjugated donkey anti-mouse IgG and HRP-conjugated goat anti-rabbit IgG were purchased from Southern Biotech, USA. Alexa Fluor 488-conjugated goat anti-mouse antibody was purchased from Abcam, UK. AbBy Fluor 488-Conjugated Goat Anti-Human IgG was purchased from Bioss Antibodies. The TruStain FcX anti-mouse CD16/32 antibody was purchased from BioLegend, USA. Mouse APC-CD19, FITC-CD40, PE-CD138, PerCP-Cy5.5-B220, APC-Cy7-CD27, PE-IgM, FITC-IgG, APC-CD80, PE-CD86, FITC-MHC I, and PE-MHC II were purchased from BD Biosciences, USA. The mouse IFN-γ/IL-4 enzyme-linked immunospot (ELISpot) kit was purchased from Dakewe Biotech (Shenzhen) Co., Ltd.

### *In vitro* transcription and lipid-nanoparticle encapsulation

Using the linearized plasmid pGEM-IVT-Gn/Gc as the template, transcription was performed with the HIScribe T7 RNA Synthesis Kit. The Cap1 structure was added to the 5′ end of the mRNA via the vaccinia virus capping enzyme and the mRNA Cap 2′-O-methyltransferase. *E. coli* poly(A) polymerase was used to add a poly(A) tail to the 3′ end of the mRNA. Pure mRNA-Gn/Gc transcripts were obtained after purification with a Monarch RNA purification kit. The transcripts were encapsulated with LNPs to obtain LNP-mRNA-Gn/Gc. The particle size of the LNP-mRNA vaccines was determined with a nanoparticle particle size analyzer.

### Protein expression

For the immunofluorescence assay, LNP-mRNA-Gn/Gc transfection was carried out according to the Lipo3000 protocol. HEK293T cells were seeded in 24-well plates and transfected with 1 µg LNP-mRNA per well for 48 h. The cells transfected with LNP-mRNA-Gc were then stained with a mouse-derived anti-SFTSV Gc polyclonal antibody and a FITC-conjugated goat anti-mouse IgG. The cells transfected with LNP-mRNA-Gn were then stained with a mouse-derived anti-SFTSV Gn monoclonal antibody and FITC-conjugated goat anti-mouse IgG. Protein expression was observed with a fluorescence microscope.

For Western blot analysis, HEK293T cells were seeded in a six-well plate, 2 µg of LNP-mRNA-Gn/Gc was transfected into HEK293T cells via Lipo3000, and the cells were collected after 24 h. The proteins were denatured by boiling, separated on a 12% SDS-PAGE gel, and then transferred to PVDF membranes. The membranes were blocked in 5% skim milk for 1 h, followed by overnight incubation at 4°C with a rabbit anti-SFTSV Gc polyclonal antibody and a mouse anti-SFTSV Gn monoclonal antibody. The membranes were subsequently washed three times with TBST (0.1% Tween-20 in TBS). The membranes were then incubated for 1 h with HRP-conjugated goat anti-rabbit IgG and HRP-conjugated donkey anti-mouse IgG and washed three times with TBST after incubation. After the PVDF membrane was reacted with an enhanced chemiluminescence reagent, the target protein bands were observed with an ultrasensitive multifunction chemiluminescence imager.

### Immunization procedure and mRNA vaccine toxicity analysis

Groups of female BALB/c mice (6–8 weeks, eight mice per group) were inoculated via intramuscular injection (i.m.) with LNP-mRNA-Gn, LNP-mRNA-Gc, or PBS. A total of 10 µg of LNP-mRNA in 100 µL PBS was injected into each mouse of the experimental groups, which were boosted with the same dose 14 days after the prime immunization. The control group received 100 µL PBS for both the prime and booster immunizations. Body weights, mental state, and dietary status were recorded daily for 21 days postimmunization.

Fifteen 1-year-old female beagles were included in the study and were randomly divided into three groups. Three dogs in the control group were intramuscularly injected with PBS. Six dogs were intramuscularly immunized with 100 µg LNP-mRNA-Gc, while the remaining six dogs were intramuscularly immunized with 100 µg LNP-mRNA-Gn. Booster immunization was administered 3 weeks after the prime immunization. The body weights and temperatures of the dogs were recorded, and blood samples were collected (body weight change rate = body wt after immunization / initial body weight × 100%).

### Serum neutralization assay

Serum samples were isolated and heat-inactivated, and SFTSV-specific neutralizing antibody titers were measured using a fluorescent reduction neutralization test (FRNT). Briefly, SFTSV (1 × 10^2^ TCID_50_) was mixed with an equal volume of serially diluted serum and incubated at 37°C for 90 min. The mixture was then inoculated onto confluent monolayers of Vero cells in 96-well plates, with virus-only infection controls included in parallel. After 2 h of incubation at 37°C with 5% CO_2_, the medium was removed and replaced with DMEM supplemented with 2% FBS and 1% antibiotics. The cells were cultured for an additional 48 h. For detection, mouse anti-SFTSV Gc polyclonal antibody was used to detect neutralizing antibodies in mouse serum, whereas human anti-SFTSV N monoclonal antibody was used for beagle serum samples. The cells were then incubated with Alexa Fluor 488-conjugated goat anti-mouse IgG and AbBy Fluor 488-Conjugated Goat Anti-Human IgG secondary antibodies, respectively. Fluorescent spots were counted under a fluorescence microscope. When the number of fluorescent spots in the sample wells was reduced by 50% compared with that in the virus control wells, the corresponding serum dilution was defined as the neutralizing antibody titer (35).

### Mice IFNAR antibody treatment and challenge infection

An IFNAR-blocked mouse model was used to assess the protective efficacy of LNP-mRNA-Gn/Gc (36). Wild-type female BALB/c mice were randomly divided into three groups and immunized with 10 µg of LNP-mRNA-Gn, LNP-mRNA-Gc, or an equal volume of PBS. At 27 days post primary immunization, each mouse received 1.5 mg of anti-mouse IFNAR-1 antibody (product no.: I-401, Leinco Technologies) via intraperitoneal injection, and then challenged with 1 × 10^5.25^ TCID_50_ of SFTSV (JS2011-013-1 strain) via the i.m. route 24 h later. The body weight, food intake, mental state, and clinical signs of the challenged mice were assessed daily for 3 weeks after infection. The clinical symptom scores and mortality rates were recorded, and survival curves were drawn. Pathology of the spleens of dead mice was performed to observe pathological changes after SFTSV infection.

### Flow cytometry

Wild-type female BALB/c mice (6–8 weeks) were randomly divided into three groups (15 mice per group; 5 mice per group per time point) and immunized with LNP-mRNA-Gn, LNP-mRNA-Gc, or the same volume of PBS. A single dose of LNP-mRNA (10 µg) in 100 µL of PBS was intramuscularly injected into each mouse in the experimental groups. At 3, 6, and 9 days postimmunization, single-cell suspensions of inguinal lymph nodes (LNs) were harvested and stained with antibodies against dendritic cell (DC) markers (CD16/32^+^, CD11c^+^, CD80^+^, CD86^+^, MHC I^+^, and MHC II^+^) at 4°C for 30 min. In addition, single lymphocytes in the spleen were isolated at 2, 4, and 8 weeks postimmunization and stained with antibodies against B cells markers (CD16/32^+^, CD19^+^, CD138^+^, CD27^+^, B220^+^, IgG^+^, and IgM^+^). The stained cells were analyzed with a Cytoflex flow cytometer (Beckman), and data analysis was performed with CytExpert 2.3 software.

### ELISpot assay

Groups of female BALB/c mice (wild type; 6–8 weeks; eight mice per group; four mice per group per time point) were inoculated i.m. with LNP-mRNA-Gn, LNP-mRNA-Gc, or the same volume of PBS. The induction of antigen-specific T cells was determined via an ELISpot assay. Mouse splenocytes were isolated at 4 and 8 weeks after the prime immunization and stimulated with 2 μg of purified inactive SFTSV for 20 h at 37°C. Secreted IFN-γ- and IL-4-specific T lymphocytes were detected via a mouse IFN-γ/IL-4 ELISpot kit (Dakewe Biotech Co. Ltd., China) following the manufacturer’s instructions and measured via a plate reader (AID ELISpot reader-iSpot, AID GmbH, and GER). The background response was subtracted when the number of spot-forming cells (SFCs) was calculated.

### ELISA

ELISA plates were coated with 800 ng/well of SFTSV Gn and Gc protein overnight at 4°C. One hundred microliters of 10-fold serially diluted serum were added and incubated overnight at 4°C. The plates were incubated with HRP-conjugated goat anti-mouse IgG1 and IgG2a at a dilution of 1:500. The absorbance was measured at 405 nm via a microplate reader (Synergy 2, BioTek). A sample was deemed positive if the absorbance reading was at least 2.1 times greater than that of the mock group. The positive antibody endpoint titers are expressed as the reciprocals of the highest dilution of serum.

### Statistical analysis

All analyses were performed with GraphPad Prism software version 8. Statistical significance was determined using Student’s *t*-test between two groups or one-way ANOVA followed by Tukey’s multiple comparisons test. The normality of data distribution was assessed using the Shapiro-Wilk test. The error bars in the figures represent the means ± SEMs, and *P* < 0.05 was considered statistically significant (*P < 0.05, **P < 0.01, ***P < 0.001, ****P < 0.0001; ns, not statistically significant).

## RESULTS

### Construction and characterization of LNP-mRNA-Gn and LNP-mRNA-Gc vaccines

**Fig 1:**
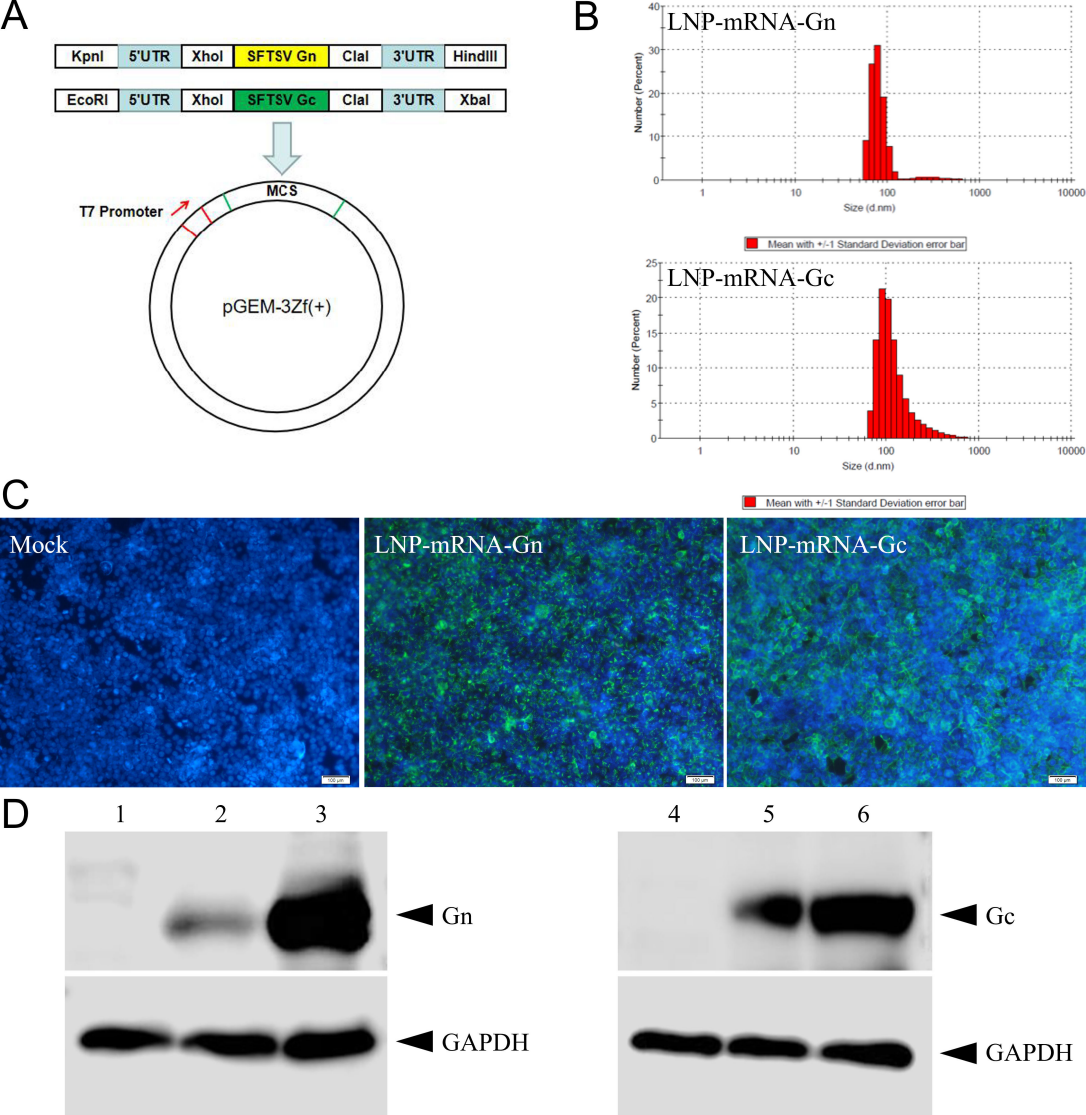
Construction and characterization of the mRNA vaccines. (**A**) Schematic diagram of the SFTSV Gn and Gc mRNA vaccines. (**B**) Particle size of LNP-mRNA. (**C**) HEK293T cells were transfected with LNP-mRNA-Gn or LNP-mRNA-Gc. Green fluorescence represents Gn or Gc, and DAPI (blue fluorescence) was used to indicate the total number of cells (scale bar = 100 µm). (**D**) HEK293T cells were transfected with LNP-mRNA-Gn or LNP-mRNA-Gc; the SFTSV-infected group served as the positive control, and the mock-transfected group served as the negative control. The expression of Gn or Gc was confirmed by immunoblot analysis. A mouse anti-SFTSV Gn monoclonal antibody and a rabbit anti-SFTSV Gc polyclonal antibody were used to detect the expression of SFTSV Gn and Gc, respectively (1: mock; 2: LNP-mRNA-Gn; 3: SFTSV; 4: mock; 5: LNP-mRNA-Gc; and 6: SFTSV).

To construct vaccine candidates, *in vitro*-transcribed mRNAs encoding Gn or Gc glycoproteins of the SFTSV strain JS2011-013-1 (GenBank: KC505127.1) were synthesized via optimized untranslated regions derived from the human β-globin gene to increase translational efficiency. The plasmids used for transcription were generated by ligating mRNA sequences into a pGEM vector (Fig. 1A). The resulting mRNAs were encapsulated in LNPs to form LNP-mRNA-Gn and LNP-mRNA-Gc, with average particle diameters of 90.2 nm and 127.3 nm, respectively, as measured by dynamic light scattering (Fig. 1B).

To verify antigen expression, HEK293T cells were transfected with the LNP-mRNA constructs and analyzed by immunofluorescence and Western blot. Distinct green fluorescence signals were observed in cells stained with anti-Gn or anti-Gc antibodies, indicating successful protein expression (Fig. 1C). Immunoblotting further confirmed the expression of the expected proteins, with bands corresponding to Gn (~61 kDa) and Gc (~55 kDa) observed in vaccine-transfected cells (Fig. 1D). These data validate the successful formulation and cellular translation of both mRNA vaccines.

### Protective efficacy and neutralizing antibody responses in mice

**Fig 2:**
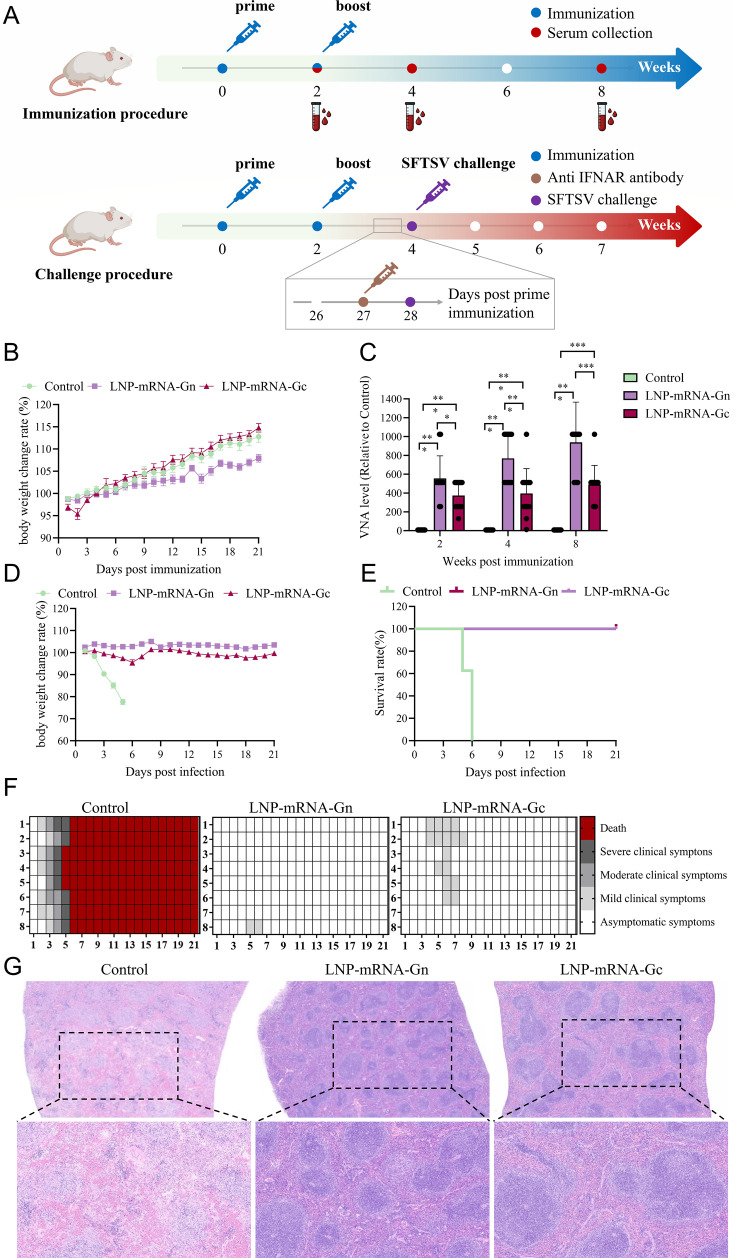
Efficacy of the mRNA vaccines in mice. (**A**) Schematic diagram of the immunization protocol, blood collection time points, and SFTSV challenge schedule in mice. (**B**) Body weight was monitored daily for 21 days postimmunization (eight wild-type mice per group; values are means ± SEM). (**C**) The specific VNAs from mouse blood samples against SFTSV were detected at 2, 4, and 8 weeks postimmunization (12 wild-type mice per group; values are means ± SEM; ANOVA with Tukey’s multiple comparisons test). (**D**) The body weights of the IFNAR-blocked mice were monitored daily for 21 days after the lethal SFTSV challenge (eight IFNAR-blocked mice per group; values are means ± SEM). (**E**) Survival curves of IFNAR-blocked mice were monitored daily for 21 days after the lethal SFTSV challenge (eight IFNAR-blocked mice per group). (**F**) Clinical symptom scores of the IFNAR-blocked mice were monitored daily after the lethal SFTSV challenge. (**G**) The spleens of vaccinated (LNP-mRNA-Gn/Gc) and unvaccinated mice were collected for histopathological examination (scale bar = 200 µm). *P < 0.05, **P < 0.01 and ***P < 0.001.

The immunization protocol and SFTSV challenge schedule for the mice were summarized in Fig. 2A. Female BALB/c mice received intramuscular injections of LNP-mRNA-Gn, LNP-mRNA-Gc, or PBS, followed by a homologous booster on day 14. No significant changes in body weight, behavior, or feeding were observed during the 21 days postimmunization, indicating a favorable safety profile of both vaccines (Fig. 2B). Serum virus-neutralizing antibody (VNA) titers were measured by FRNT at 2, 4, and 8 weeks after prime immunization in immunocompetent BALB/c mice to evaluate antibody persistence. Both vaccines elicited durable neutralizing antibody responses. However, LNP-mRNA-Gn consistently induced significantly higher titers than LNP-mRNA-Gc did across all time points (mean titer of SFTSV VNA at week 8: 938 vs 490; Fig. 2C), demonstrating its superior humoral immunogenicity in mice.

Protective efficacy was assessed in an IFNAR-blocked mouse model challenged with a lethal dose of SFTSV 4 weeks after prime immunization. Both the LNP-mRNA-Gn and LNP-mRNA-Gc vaccines provided complete protection, with 100% survival and only mild, transient symptoms such as minor weight loss or ruffled fur (Fig. 2D through F). In contrast, all PBS-treated control mice presented severe disease manifestations, including significant weight loss, lethargy, and 100% mortality. Histological examination of the spleens revealed no abnormal pathology in the vaccinated animals, whereas the spleens from the control mice displayed necrosis of white pulp and disorganized splenic architecture (Fig. 2G).

### Induction of innate and adaptive immune responses in mice

**Fig 3:**
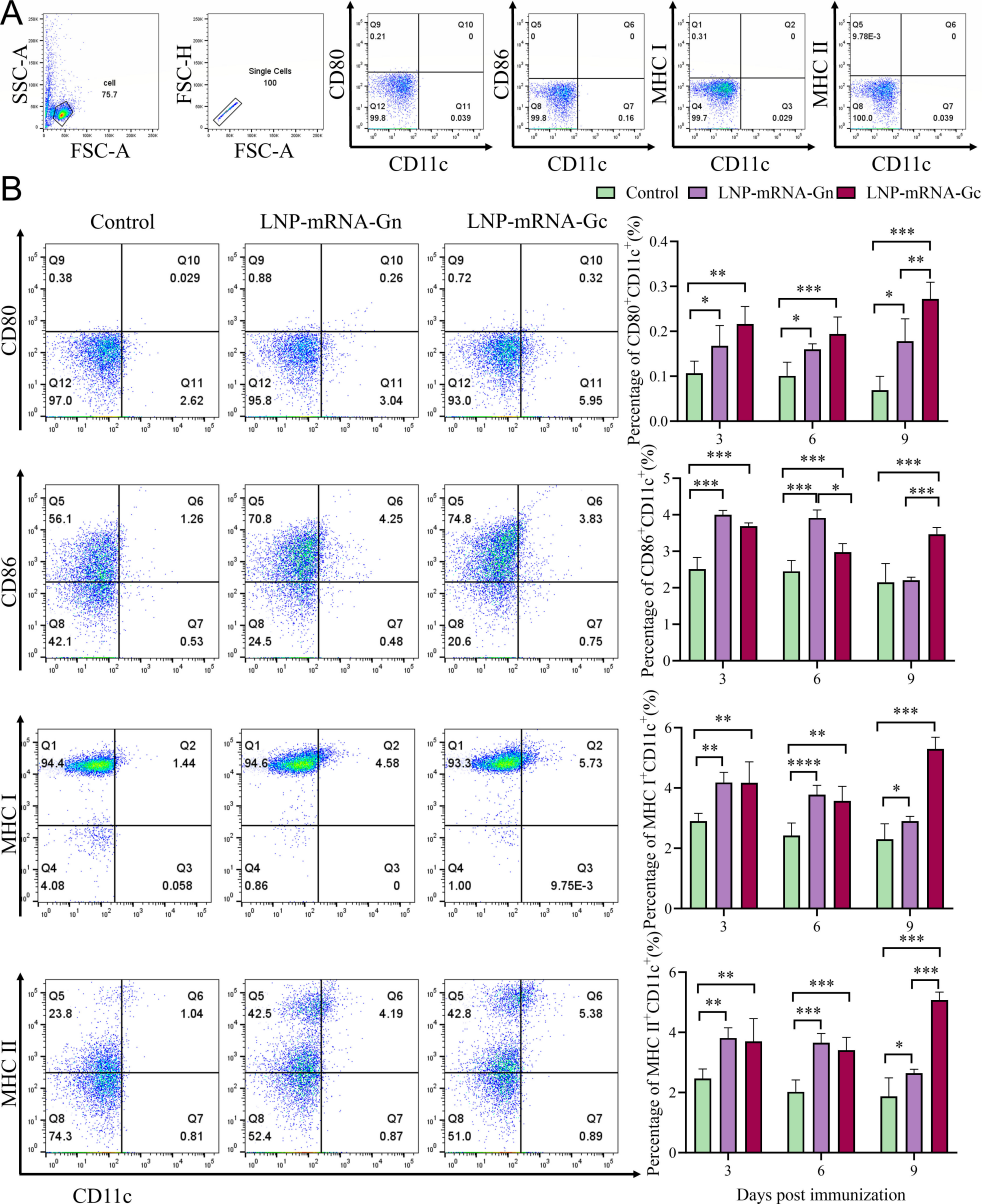
Activation of DCs in lymph nodes. (**A**) Gating strategies for analyzing DCs. (**B**) Inguinal LNs were collected at 3, 6, and 9 days postimmunization. Single-cell suspensions were prepared and stained separately with antibodies against DCs (CD11c^+^, CD80^+^, CD86^+^, MHC I^+^, and MHC II^+^). A representative flow cytometric plot of activated DCs is shown. Statistical analysis of the percentages of activated DCs in the lymph nodes (15 wild-type mice per group; 5 mice per group per time point; values are means ± SEM; ANOVA with Tukey’s multiple comparisons test). *P < 0.05, **P < 0.01 and ***P < 0.001.

To investigate early immune activation, DC responses in inguinal lymph nodes were analyzed via flow cytometry at 3, 6, and 9 days postimmunization. The gating strategy is shown in Fig. 3A. Compared with the PBS controls, both LNP-mRNA-Gn and LNP-mRNA-Gc significantly increased the proportion of activated DCs expressing CD80, CD86, MHC I, and MHC II (Fig. 3B), indicating robust initiation of innate immune responses.

**Fig 4:**
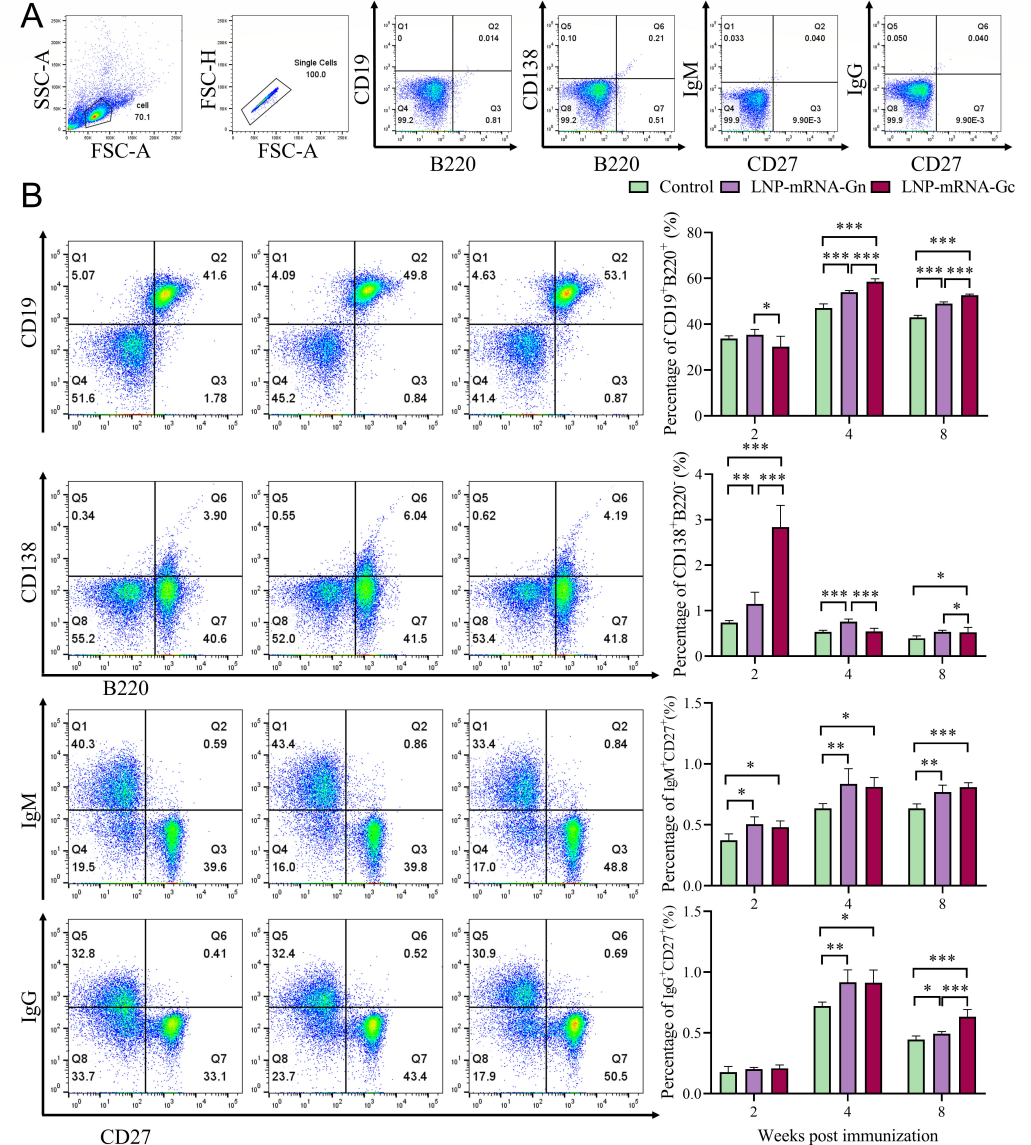
Activation of B cells in the spleen. (**A**) Gating strategies for analyzing B cells. (**B**) Spleens were collected at 2, 4, and 8 weeks postimmunization. Single-cell suspensions were prepared and stained separately with antibodies against B cells (CD19^+^, CD138^+^, CD27^+^, B220^+^, IgG^+^, and IgM^+^). A representative flow cytometric plot of activated B cells is shown. Statistical analysis of the percentages of activated B cells in the spleen (15 wild-type mice per group; 5 mice per group per time point; values are means ± SEM; ANOVA with Tukey’s multiple comparisons test). *P < 0.05, **P < 0.01 and ***P < 0.001.

For humoral immune profiling, splenic B-cell subsets were analyzed at 2, 4, and 8 weeks postimmunization. The gating strategy is presented in Fig. 4A. Compared with control mice, mice immunized with either vaccine presented significant increases in CD19^+^B220^+^ B cells, memory B cells (CD27^+^IgM^+^ and CD27^+^IgG^+^), and plasma cells (CD138^+^B220^−^) (Fig. 4B), reflecting potent and sustained B-cell activation.

**Fig 5:**
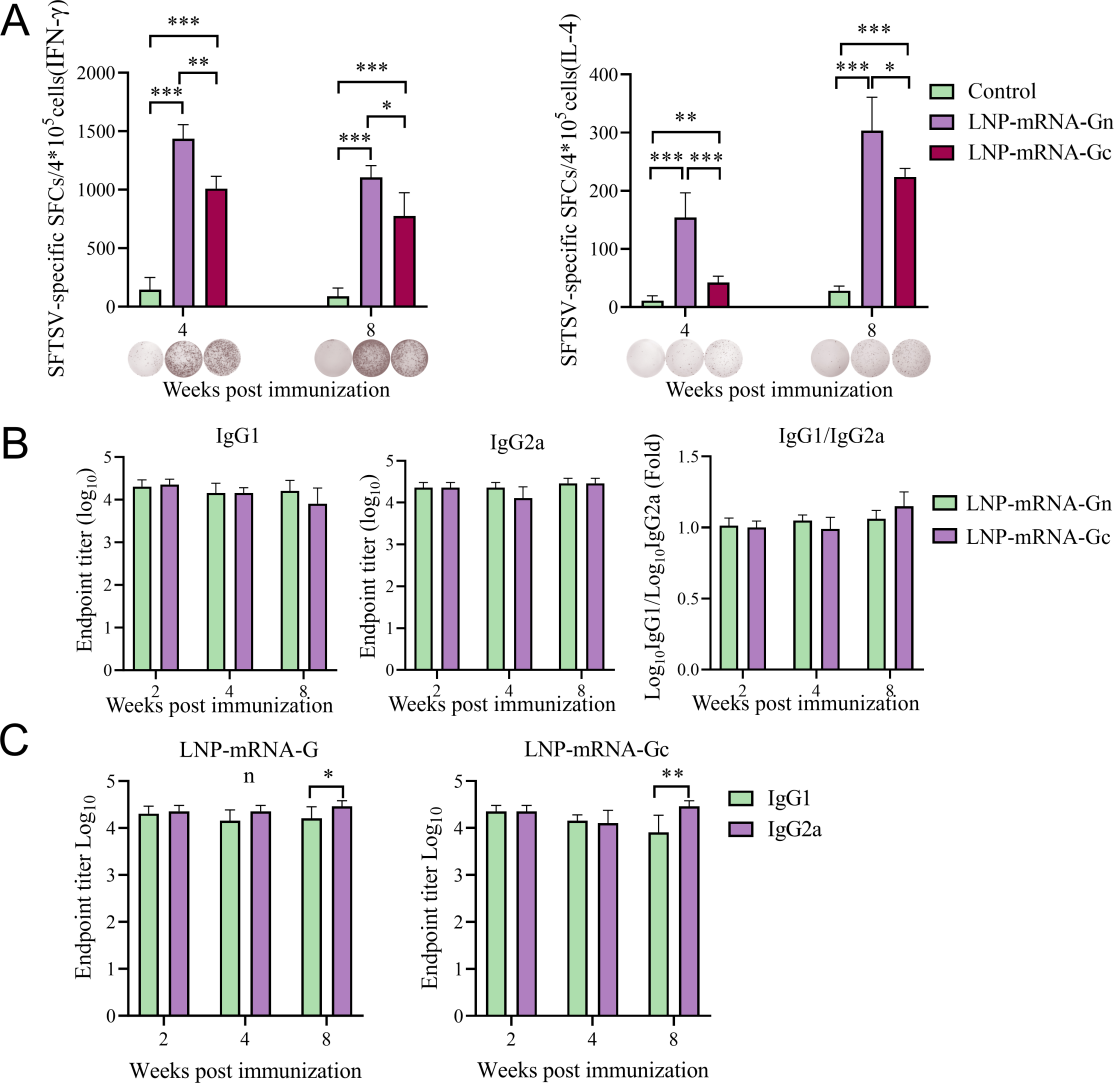
Activation of the Th1/Th2-mediated immune response after LNP-mRNA-Gn/Gc immunization. (**A**) Inactivated purified SFTSV virion-specific IFN-γ^+^ SFCs and IL-4^+^ SFCs were detected via ELISpot kits (eight wild-type mice per group; four mice per group per time point; values are means ± SEM; ANOVA with Tukey’s multiple comparisons test). (**B, C**) The relative ratios of SFTSV-specific IgG1, IgG2a, and IgG1/IgG2a in LNP-mRNA-Gn- and LNP-mRNA-Gc-immunized mice (18 wild-type mice per group; 6 mice per group per time point; values are means ± SEM; Student’s *t*-test). *P < 0.05, **P < 0.01 and ***P < 0.001.

T-cell functionality was evaluated via ELISpot at 4 and 8 weeks postimmunization. Both vaccine groups presented elevated frequencies of IFN-γ- and IL-4-secreting splenocytes, confirming the activation of both the Th1 and Th2 subsets (Fig. 5A). Notably, LNP-mRNA-Gn induced significantly more cytokine-producing T cells than LNP-mRNA-Gc did, supporting its enhanced cellular immunogenicity.

To further assess immune polarization, IgG1 and IgG2a titers were measured. Both vaccines elicited balanced Th1/Th2 responses at early time points, with a shift toward Th1 dominance (higher IgG2a/IgG1 ratio) at week 8 (Fig. 5B and C). These data indicate that both mRNA vaccines activate a broad adaptive immune response, with LNP-mRNA-Gn promoting stronger T-cell and antibody responses in mice.

### Immunogenicity of LNP-mRNA-Gn and LNP-mRNA-Gc in dogs

**Fig 6:**
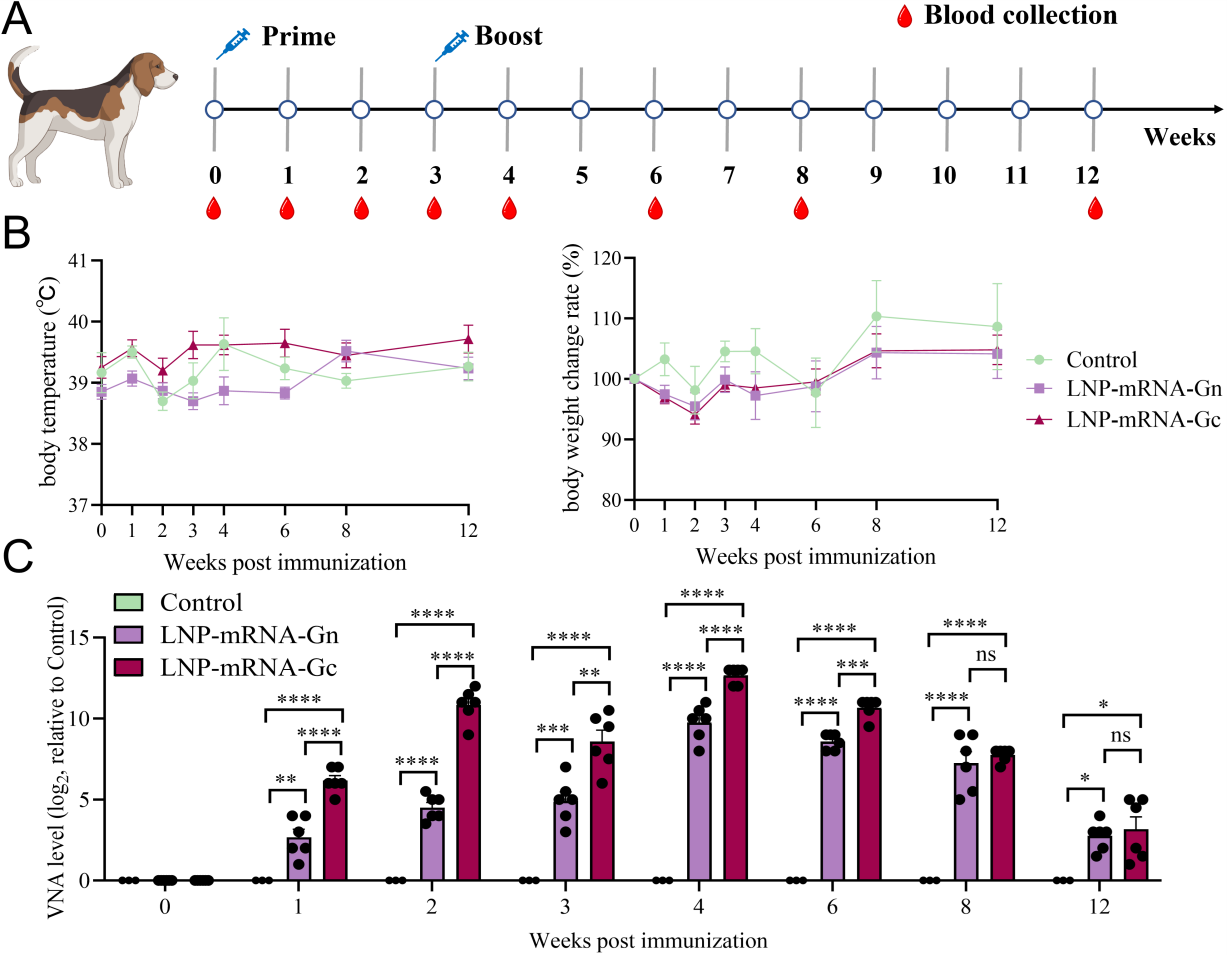
Efficacy of mRNA vaccines in dogs. (**A**) Schematic diagram of the immunization procedure for the dogs and the blood collection time point. (**B**) Body temperatures and weights of the dogs were recorded at the time of each blood sample collection. (**C**) The specific VNAs from dog blood samples against SFTSV were detected at 0, 1, 2, 3, 4, 6, 8, and 12 weeks postimmunization (control group [*n* = 3]; LNP-mRNA-Gn group [*n* = 6]; LNP-mRNA-Gc group [*n* = 6]; values are means ± SEM; ANOVA with Tukey’s multiple comparisons test). *P < 0.05, **P < 0.01, ***P < 0.001, ****P < 0.0001; ns, not statistically significant.

To explore species-specific immunogenicity, we assessed vaccine responses in beagle dogs, a relevant companion animal host. The immunization strategy and sampling schedule are outlined in Fig. 6A. Dogs received 100 µg of LNP-mRNA-Gn, LNP-mRNA-Gc, or the same volume of PBS via intramuscular injection, with a booster administered 3 weeks later.

Throughout the 12 week observation period, no significant differences in body temperatures or weights were noted between the vaccinated and control animals (Fig. 6B). Complete blood count profiles remained within normal ranges for all groups, indicating excellent safety and tolerability (Fig. S1).

SFTSV-neutralizing antibody titers were measured at multiple time points. Interestingly, the immunogenic hierarchy observed in mice was reversed in dogs: LNP-mRNA-Gc elicited significantly higher neutralizing antibody titers than LNP-mRNA-Gn did, with peak responses observed between weeks 4 and 8 (Fig. 6C). After week 8, the titers declined and converged between the two groups. These results highlight a clear species-specific difference in vaccine immunogenicity and suggest that antigen dominance for SFTSV is not conserved across hosts. In dogs, the neutralizing antibody titers observed in this study reflect immunogenicity only and should not be interpreted as evidence of protective efficacy.

Overall, both mRNA vaccines are immunogenic and well tolerated in dogs, but the observed divergence in antigen responsiveness emphasizes the need for host-specific optimization in zoonotic vaccine design.

## DISCUSSION

Despite the increasing recognition of companion animals as potential reservoirs and amplifiers of SFTSV (22–24), no veterinary vaccine is currently available. Here, we report the development of two LNP-encapsulated mRNA vaccines encoding the Gn and Gc glycoproteins of SFTSV and evaluate their immunogenicity across species. Our findings highlight a previously unreported divergence in antigen-specific responses between murine and canine models, underscoring the complexity of host-adapted vaccine design.

In mice, the Gn mRNA vaccine elicited consistently stronger humoral and cellular responses than its Gc counterpart. It induced higher neutralizing antibody titers, more robust DC activation, greater recruitment of memory B cells, and enhanced Th1/Th2 cytokine production. These observations are consistent with previous studies identifying Gn as the dominant target of neutralizing antibodies, likely because of its surface exposure and epitope accessibility (10, 37). Importantly, both vaccines provided full protection in IFNAR-blocked mice upon lethal challenge, supporting their efficacy even in a more immunocompetent model than in conventional IFNAR-knockout mice. This finding also confirms that robust antibody responses are sufficient to prevent fatal SFTSV disease *in vivo*, even when generated from distinct glycoprotein antigens.

In this study, we used an mRNA-LNP platform encoding the two SFTSV envelope glycoproteins, Gn and Gc, and observed a marked reversal in neutralizing antibody dominance between mice and dogs. In mice, the Gn mRNA vaccine induced stronger immune activation, whereas in dogs, the Gc mRNA vaccine elicited higher neutralizing antibody titers. These observations suggest that antigen dominance between Gn and Gc is not fixed but may be shaped by host-specific immune determinants. Based on these findings, we propose several specific and testable mechanistic hypotheses. First, species-specific differences in antigen processing and MHC class II presentation may differentially favor Gn- or Gc-derived epitopes, thereby influencing CD4^+^ T cell help, particularly T follicular helper cell responses, and ultimately shaping germinal center output and neutralizing antibody quality. This hypothesis is consistent with our observation that Gn induced stronger immune activation in mice and could be tested by comparing Gn- and Gc-derived peptide presentation and CD4^+^ T cell responses in representative murine H-2 and canine DLA backgrounds (38, 39). Second, species-dependent differences in the naive B-cell receptor repertoire may influence which neutralizing epitopes on Gn and Gc are most readily recognized and expanded after vaccination. In this framework, the murine repertoire may preferentially focus on neutralizing determinants within Gn, whereas the canine repertoire may more readily recognize key neutralizing surfaces on Gc. This possibility could be evaluated by combining single-cell BCR repertoire analysis with epitope mapping of vaccine-induced antibodies in both species (40). Third, host-dependent differences in the *in vivo* expression, post-translational processing, or glycan-dependent epitope exposure of Gn and Gc after mRNA-LNP delivery may alter the relative accessibility of neutralizing epitopes and thereby contribute to the observed reversal of immunodominance. This hypothesis could be examined by comparing antigen expression, processing, and glycosylation patterns in murine and canine systems (41, 42). These mechanisms were not directly tested in the present study and therefore remain hypotheses. However, they provide a more biologically grounded and testable framework for interpreting the species-specific reversal of Gn/Gc immunodominance observed here and for guiding future studies on antigen selection in zoonotic vaccine design. Collectively, these hypotheses provide specific and testable mechanistic explanations for the reversal of immunodominance observed between mice and dogs in this study. These findings further emphasize that the immunodominant hierarchy of viral glycoproteins is not conserved across species and must be empirically validated in each relevant host (43, 44).

The broader implication is that relying solely on murine data to define optimal vaccine antigens may be insufficient for identifying pathogens with complex ecological niches. For SFTSV, which circulates between ticks, wild animals, domestic pets, and humans (17–19), species-specific immunogenicity becomes a key variable in achieving herd-level immunity and breaking transmission chains. Our findings support a paradigm shift toward host-adapted vaccine formulations, particularly in the context of emerging zoonoses where companion animals serve as transmission bridges.

Mechanistically, in mice, both mRNA vaccines activated key arms of the adaptive immune response. Specifically, they induced early dendritic cell maturation and sustained Th1-biased cytokine production (IFN-γ >IL-4), which is consistent with the antiviral profile required for clearance of cytoplasmic RNA viruses. Memory B-cell formation and long-lived neutralizing antibody production were observed in both vaccine groups, although Gn elicited higher titers. The LNP platform likely contributed to these responses by promoting endosomal escape and efficient antigen presentation via both the MHC I pathway and the MHC II pathway.

Both vaccines were well tolerated in both mice and dogs, with no significant adverse effects on body weights, temperatures, or hematological indices. This safety profile is consistent with that of other mRNA vaccines and supports their further development in both veterinary and human applications.

While prior research has described two SFTSV mRNA vaccine candidates utilizing Gn-head and full-length GPs as immunogens (37, 45), there have been no reported mRNA vaccines specifically designed to target the Gn and Gc proteins of SFTSV individually. Compared with full-length GP vaccines, the Gn- and Gc-based mRNA vaccines used in this study provide a basis for optimizing antigen selection in a species-specific manner.

Limitations of this study include the absence of viral challenge experiments in dogs due to ethical constraints and the lack of in-depth T-cell profiling in the canine model. Future work will need to address these gaps through the use of surrogate markers or naturally infected cohorts. In addition, an empty LNP control was not included in the immunization and toxicity analyses. Therefore, although no overt adverse effects were observed in the mRNA-LNP vaccination groups compared with the PBS group, the potential contribution of the LNP carrier itself could not be independently assessed. Inclusion of an empty LNP control in future studies will allow a more precise evaluation of LNP-associated effects. Another limitation is that we did not include the full-length GP or a combination of Gn and Gc mRNAs. However, the purpose of this study is to identify the optimal immunogenic regions of the SFTSV M protein and the differences in immunogenicity across different species. However, since the *M* segment is approximately 3,378 nt in length, which exceeds the optimal length for mRNA vaccine design (around 1,000 nt), such a long coding sequence presents challenges in structural optimization, manufacturing stability, and protein yield. Currently, we are developing a full-length M protein vaccine based on circRNA, which leverages its higher loading capacity, improved stability, and more sustained antigen expression to achieve more effective *in vivo* expression of the SFTSV M protein. A limitation of this study is the absence of an empty LNP control in the immunization and toxicity analyses, which would allow a more precise evaluation of LNP-associated effects. Moreover, mRNA vaccines remain limited by stability challenges under field conditions, which may constrain their deployment in endemic rural settings (46). Strategies to increase thermostability and simplify drug administration are essential for future field use.

In conclusion, this study provides a novel cross-species comparison of mRNA vaccine immunogenicity against SFTSV and highlights species-dependent differences in antigen dominance. Both Gn- and Gc-based mRNA vaccines confer protection in mice while eliciting distinct neutralizing antibody profiles in dogs. These findings call for broader consideration of host-specific immune responses in zoonotic vaccine design and suggest that tailored antigen selection may be required for optimal efficacy across species. Our results lay the foundation for further development of dual-use vaccines targeting both human and animal hosts for the control of emerging bunyaviral diseases.

## Data Availability

The data supporting the findings of this study are openly accessible in the Science Data Bank (47).

## SUPPLEMENTAL MATERIAL

Fig. S1 spectrum.03603-25-s0001.docx CBC data from the immunized canine.

ASM does not own the copyrights to Supplemental Material that may be linked to, or accessed through, an article. The authors have granted ASM a non-exclusive, world-wide license to publish the Supplemental Material files. Please contact the corresponding author directly for reuse.
