# Supplementary material for: Differential immune responses to SFTSV Gn and Gc mRNA vaccines in mice and dogs
Source: Microbiol Spectr. 2026 Jul 13;14(8):e03603-25. doi: 10.1128/spectrum.03603-25 (PMC13435832; doi:10.1128/spectrum.03603-25)
Supplement: Supplementary file 1 [file spectrum.03603-25-s0001.docx]

Supplementary Materials

**Differential Immune Responses to SFTSV Gn and Gc mRNA Vaccines in Mice and Dogs**

Zezheng Jiang^a#^, Li Tian^a#^, Zhongxin Zhao^b#^, Xinyu Wu^a^, Lele Liu^a^, Menghua Li^a^, Hua Qian^c^, Chunhai Gao^b^, Xiaoying Lei^a*^, Wenwen Zheng^a*^, Xuejie Yu^d*^, Xuexing Zheng^a, c*^

^a^ Department of Virology, School of Public Health, Cheeloo College of Medicine, Shandong University, Jinan, Shandong, 250012, China.

^b^ Department of Laboratory Medicine, Linyi People's Hospital, Linyi, Shandong, 276002 China.

^c^ The Second Hospital, Cheeloo College of Medicine, Shandong University, Jinan, 250033, Shandong, China.

^d^ State Key Laboratory of Virology, School of Public Health, Wuhan University, Wuhan, Hubei, China

^#^Zezheng Jiang, Li Tian and Zhongxin Zhao contributed equally to this work.

^*^Corresponding author:

Xuexing Zheng, E-mail: [zhengxx2513@hotmail.com](mailto:zhengxx2513@hotmail.com); Tel: +86-531-88382625, Fax: +86-531-88382553; Xuejie Yu, [yuxuejie@whu.edu.cn](mailto:yuxuejie@whu.edu.cn); Wenwen zheng, E-mail: zhengwenwen@sdu.edu.cn; Xiaoying Lei, leixy@sdu.edu.cn

**Fig. S1. The blood routine test results of the dog.** Peripheral blood was collected from the right forelimb of beagles using EDTA-containing blood collection tubes. WBC, LYMPH, MONO, NEUT, EO, HGB, MCH, RBC, MCV, HCT, and PLT were measured using ProCyte Dx^®^ Hematology Analyzer and compared with normal reference ranges.
